# Cooperative pathogenicity in cystic fibrosis: *Stenotrophomonas maltophilia* modulates *Pseudomonas aeruginosa* virulence in mixed biofilm

**DOI:** 10.3389/fmicb.2015.00951

**Published:** 2015-09-16

**Authors:** Arianna Pompilio, Valentina Crocetta, Serena De Nicola, Fabio Verginelli, Ersilia Fiscarelli, Giovanni Di Bonaventura

**Affiliations:** ^1^Department of Medical, Oral and Biotechnological Sciences, “G. d'Annunzio” University of Chieti-PescaraChieti, Italy; ^2^Aging Research Center (Ce.S.I.), “G. d'Annunzio” University FoundationChieti, Italy; ^3^Department of Pharmacy, “G. d'Annunzio” University of Chieti-PescaraChieti, Italy; ^4^Children's Hospital and Research Institute “Bambino Gesù”Rome, Italy

**Keywords:** cystic fibrosis, lung infections, *Stenotrophomonas maltophilia*, *Pseudomonas aeruginosa*, microbial interactions

## Abstract

The present study was undertaken in order to understand more about the interaction occurring between *S. maltophilia* and *P. aeruginosa*, which are frequently co-isolated from CF airways. For this purpose, *S. maltophilia* RR7 and *P. aeruginosa* RR8 strains, co-isolated from the lung of a chronically infected CF patient during a pulmonary exacerbation episode, were evaluated for reciprocal effect during planktonic growth, adhesion and biofilm formation onto both polystyrene and CF bronchial cell monolayer, motility, as well as for gene expression in mixed biofilms. *P. aeruginosa* significantly affected *S. maltophilia* growth in both planktonic and biofilm cultures, due to an inhibitory activity probably requiring direct contact. Conversely, no effect was observed on *P. aeruginosa* by *S. maltophilia*. Compared with monocultures, the adhesiveness of *P. aeruginosa* on CFBE41o- cells was significantly reduced by *S. maltophilia*, which probably acts by reducing *P. aeruginosa's* swimming motility. An opposite trend was observed for biofilm formation, confirming the findings obtained using polystyrene. When grown in mixed biofilm with *S. maltophilia, P. aeruginosa* significantly over-expressed *aprA*, and *algD*—codifying for protease and alginate, respectively—while the quorum sensing related *rhlR* and *lasI* genes were down-regulated. The induced alginate expression by *P. aeruginosa* might be responsible for the protection of *S. maltophilia* against tobramycin activity we observed in mixed biofilms. Taken together, our results suggest that the existence of reciprocal interference of *S. maltophilia* and *P. aeruginosa* in CF lung is plausible. In particular, *S. maltophilia* might confer some selective “fitness advantage” to *P. aeruginosa* under the specific conditions of chronic infection or, alternatively, increase the virulence of *P. aeruginosa* thus leading to pulmonary exacerbation.

## Introduction

Pulmonary disease is the leading cause of morbidity and mortality in cystic fibrosis (CF) patients, in whom defective mucociliary clearance and impaired innate immunity lead to chronic pulmonary infections (Lyczak et al., 2002). During CF airway disease periods of stability are punctuated by acute pulmonary exacerbations (PEs) in which overt immunological responses are the main causes of irreversible lung damage (Amadori et al., 2009; Sanders et al., 2011). Recurrent PEs are associated with a shortened survival (Emerson et al., 2002; Marshall, 2004).

The pathophysiology of PEs is not yet completely understood. Aside from acute viral infections which, especially in children, have been associated with up to one-third of PEs (Armstrong et al., 1998; Clifton et al., 2008), *Pseudomonas aeruginosa* has been considered the primary cause of PEs and related long-term decline in lung function (Goss and Burns, 2007; Sanders et al., 2010).

The pathogenesis of *P. aeruginosa* infection depends on several cell-associated and extracellular virulence factors, including proteases and toxins, whose expression is mainly regulated by hierarchically organized inter-bacterial communication LasRI and RhIRI quorum sensing (QS) systems, which monitor population size using various diffusible N-acylhomoserine lactones as signal molecules (Goodman and Lory, 2004).

However, this view is not convincingly supported by either the clinical or the microbiological evidence available.

Several studies have reported that in adult CF patients the anti-pseudomonal antibiotic therapy frequently did not reduce *P. aeruginosa* load and airways inflammation (Wolter et al., 1999; Reid et al., 2004), and that about 25% of patients experiencing a PE did not recover their baseline lung function after treatment, leading to a progressive deterioration in their clinical status over time (Sanders et al., 2010, 2011). Furthermore, in other studies no increase in *P. aeruginosa* concentration was observed immediately prior to, or at the time of, PE (Stressmann et al., 2011; Reid et al., 2013). Recent epidemiological data indicate that coinfections involving different species of bacteria are common, and probably represent the norm, in CF lung (Harrison, 2007; Bittar et al., 2008; Sibley et al., 2008a; Rogers et al., 2010a). *P. aeruginosa* is the most common species found in CF airways, but other species are frequently co-isolated in CF lung (Harrison, 2007).

The pathophysiology of PEs in CF could be, therefore, directly related to changes in microbial behavior and/or to the dynamics of the interactions between the constituents of the complex microbial communities present. Several studies have recently highlighted the potential role of interspecies interactions in influencing infection status, clinical outcomes or response to therapy in CF patients (Harrison, 2007; Ryan et al., 2008; Sibley et al., 2008b, 2009; Shank and Kolter, 2009; Rogers et al., 2010b). Taken together, these findings suggest that the role of microbial species other than *P. aeruginosa* needs to be considered.

*Stenotrophomonas maltophilia* is one of the most common emerging multi-drug resistant organisms found in the lungs of people with CF where its prevalence is increasing (Ciofu et al., 2013). Nevertheless, the role of *S. maltophilia* in the pathogenesis of CF lung disease is not yet clear because of conflicting results from clinical studies which focused on the correlation between the presence of this microorganism and lung damage (Karpati et al., 1994; Goss et al., 2002). In a series of studies, we found evidence highly suggestive of the pathogenic role of *S. maltophilia* in CF patients (Di Bonaventura et al., 2004, 2007a,b, 2010; Pompilio et al., 2008, 2010, 2011). This microorganism can grow as biofilms—sessile communities inherently resistant to antibiotics and host immune response—not only on abiotic surfaces (Di Bonaventura et al., 2004, 2007a,b; Pompilio et al., 2008), but also on CF-derived epithelial monolayer (Pompilio et al., 2010), probably because of a selective adaptation to CF airways (Pompilio et al., 2011). Furthermore, in a murine model of acute respiratory infection we observed that *S. maltophilia* significantly contributes to the inflammatory process resulting in compromised respiratory function and death (Di Bonaventura et al., 2010).

*S. maltophilia* is often co-isolated with *P. aeruginosa*, with a frequency ranging from 10 to 60% of CF patients (Spicuzza et al., 2009; Blau et al., 2014; Zemanick et al., 2015); however, no data are available on this at the onset of PE. It is, therefore, plausible to hypothesize that these species interact and that this could potentially affect the virulence and persistence of *P. aeruginosa*. While several studies have focused on the interaction of *P. aeruginosa* with other bacterial species (Qin et al., 2009; Pihl et al., 2010; Baldan et al., 2014), very little has been published on the interaction between *P. aeruginosa* and *S. maltophilia*. In this regard, Ryan et al. (2008) found that the presence of *S. maltophilia* increases *P. aeruginosa* resistance to polymyxin by a diffusible signal factor, while Kataoka et al. (2003) observed that β-lactamases leaking from *S. maltophilia* can encourage the growth of *P. aeruginosa* in the presence of imipenem or ceftazidime.

Recently, we observed that the pre-incubation of the CF bronchial epithelial IB3-1 cells with *S. maltophilia* decreases the adherence of *P. aeruginosa*, while previous *P. aeruginosa* infection may increase the likelihood of *S. maltophilia* colonizing a damaged CF pulmonary mucosa (Pompilio et al., 2010).

Our hypothesis, based on these clinical and experimental findings, was that in CF lung *S. maltophilia* may indirectly contribute to disease development by providing a favorable growth environment for *P. aeruginosa* and/or by modulating some virulence traits exhibited by *P. aeruginosa*. In order to test this hypothesis, *S. maltophilia* RR7 and *P. aeruginosa* RR8—two strains co-isolated at the same time from the lung of a CF patient during a PE episode—were evaluated, both in combined and monomicrobial cultures, with respect to planktonic growth, adhesion and biofilm formation onto both polystyrene and CF bronchial cell monolayer, and motility. The effect of *S. maltophilia* RR7 on the expression of selected *P. aeruginosa* RR8 virulence genes was also assessed in mixed biofilms.

We show that *S. maltophilia* has the potential to significantly affect *P. aeruginosa* virulence, which suggests that interspecies interactions are operative in promoting bacterial pathogenicity in CF lung infections.

## Materials and methods

### Bacterial strains and growth conditions

*P. aeruginosa* RR8 and *S. maltophilia* RR7 strains were co-isolated from the same sputum sample collected, during a PE, from a 15-year-old CF patient (named “RR”) attending Children Hospital “Bambino Gesù” of Rome. The patient was selected because he was chronically infected by both *P. aeruginosa* and *S. maltophilia*, according to the definition proposed by the EuroCareCF Working Group (Pressler et al., 2011). The strains were identified by the API 20-NE system (bioMérieux, Marcy-L'Etoile, France) and stored at −80°C until use. Fresh bacterial stocks were thawed and grown twice on Mueller-Hinton agar (MHA; Oxoid S.p.A., Garbagnate M.se, Italy) to check for purity and to regain the original phenotype: (i) *P. aeruginosa* RR8 strain was mucoid and resistant to amikacin and gentamicin only; (ii) *S. maltophilia* RR7 strain, not mucoid, showed resistance to ceftazidime, piperacillin/tazobactam, amikacin, gentamicin, ciprofloxacin, and levofloxacin.

All assays were carried out by using a standardized bacterial inoculum. Briefly, an overnight culture in tryptone soya broth (TSB), grew under agitation (130 rpm) at 37°C, was adjusted with sterile broth to an OD_550_ corresponding to 1–3 × 10^8^ CFU/ml for both strains, then diluted 1:10 in cation-adjusted Mueller-Hinton broth (CAMHB; Becton, Dickinson and Company; Milan, Italy) (pH 7.2–7.4).

### Mono- and co-culture planktonic growth

Each well of a 96-well polystyrene, flat bottom, tissue culture-treated microtiter (BD Falcon, Milan, Italy) was seeded with 100 μl of each standardized inoculum (dual cultures) or 100 μl of standardized inoculum +100 μl sterile medium (single cultures). Microtiters were then statically incubated at 37°C, under aerobic atmosphere, for 24 h. At different time points (2, 4, 6, 8, 10, and 24 h) samples were taken, serially diluted in sterile phosphate buffered saline (PBS; Sigma-Aldrich S.p.A.; Milan, Italy) (pH 7.2), then plated onto MHA + imipenem at 32 μg/ml or *Pseudomonas* cetrimide agar (Oxoid) to discriminate *S. maltophilia*, and *P. aeruginosa* growth, respectively. The agar plates were incubated at 37°C for 24 h when the CFU number was recorded.

### Kinetics of adhesion and biofilm formation by single and mixed cultures

Each well of a 96-well polystyrene, flat bottom, tissue culture-treated microtiter was seeded as described above, and statically incubated at 37°C, under aerobic atmosphere, for 3 h (adhesion) or up to 7 days (biofilm formation). During biofilm formation assay, CAMHB was replaced daily with fresh broth. At each time-point considered (3 h, and 1–7 days), samples were washed twice with sterile PBS, and then adhesion or biofilm were measured for both biomass and viability. Biomass was measured in terms of optical density read at 492 nm (OD_492_) following crystal violet staining, as previously described (Pompilio et al., 2008), considering a low cut-off of ODc + 3 × SDs, where ODc is OD_492_ of control wells (containing medium alone without bacteria). For viability assessment, samples were exposed to trypsin-ethylenediaminetetraacetic acid (EDTA) 0.25% (Sigma-Aldrich) to allow detachment from the polystyrene, and then the viable count was carried out as described above.

### Preparation of cell-free culture supernatant

For each strain, some colonies from an overnight MHA growth were suspended in 20 ml of TSB and incubated overnight at 37°C, under agitation (220 rpm). Supernatants were obtained after centrifugation at 12,000 × g, for 10 min at 4°C, followed by sterile filtration using a 0.20 μm-pore-size filter (Corning; Tewksbury, MA, USA), and storage at −80°C until use.

### Evaluation of antibacterial activity by *S. maltophilia* RR7 and *P. aeruginosa* RR8

The antibacterial activity of each strain was evaluated by the drop test assay. Briefly, 100 μl of the standardized inoculum containing the indicator strain were streaked onto an MHA surface using a cotton swab, and then the plate was dried at 30°C for 30 min. The test strain was then evaluated for antimicrobial activity, both as cell suspension and supernatant. Drops containing 10 μl of the supernatant or standardized suspension, and a control, were placed on the agar surface and the inhibition zone diameter was measured after incubation at 37°C for 24 h.

To evaluate whether the inhibitory activity of *P. aeruginosa* RR8 on *S. maltophilia* RR7 required direct cell-cell interactions, a transwell-based assay was performed. Briefly, *P. aeruginosa* RR8 was inoculated at 1–5 × 10^8^ CFU/ml in the upper insert (Cell Culture Insert; BD Falcon), while *S. maltophilia* RR7 was inoculated at the same concentration in the bottom of the culture well. Following 24 h-incubation at 37°C, suspensions from both compartments underwent a viable count. A sample where the upper insert contained TSB without *P. aeruginosa* RR8 was used as control.

### Adhesion to, and biofilm formation on CF bronchial cells

CFBE41o- bronchial cells, derived from a CF patient homozygous for the F508del CFTR mutation, were obtained from Dr. Dieter C. Gruenert (University of California, San Francisco, USA). Briefly, confluent CFBEo- cells were grown in tissue polystyrene culture flasks (BD Falcon) in Minimum Essential Medium (GIBCO, Life-technologies; Monza, Italy) supplemented with 10% fetal bovine serum (GIBCO), 50 U/ml penicillin, 50 μg/ml streptomycin (both from Sigma-Aldrich), and 2 mM L-glutamine (GIBCO). Monolayers were simultaneously infected with *S. maltophilia* RR7 and *P. aeruginosa* RR8 strains (ratio 1:1, each at 10^6^ CFU/ml; multiplicity of infection, MOI: 10), then incubated at 37°C for 3 h (adhesion assay) or 24 h (biofilm formation assay). CFBE41o- cell monolayers infected with a single strain (at 10^6^ CFU/ml) were prepared as control. At the end of incubation, infected monolayers were washed twice with PBS, then detached by 0.25% trypsin/EDTA, and finally plated for colony count.

### Tobramycin activity against planktonic and biofilm cells

MICs of tobramycin (Sigma-Aldrich) for *S. maltophilia* RR7 and *P. aeruginosa* RR8 were respectively 128 and 4 μg/ml, as assessed by microdilution technique in accordance with the M100-S20 protocol [Clinical and Laboratory Standards Institute, 2010]. Tobramycin was also tested against preformed single species and mixed biofilms. Briefly, biofilms were allowed to form at 37°C for 24 h as described in “Kinetics of adhesion and biofilm formation by single and mixed cultures.” Samples were washed once with sterile CAMHB, and then exposed to 200 μl of tobramycin at 128 μg/ml. After incubation at 37°C for 24 h, non-adherent bacteria were removed by washing twice with sterile PBS, and biofilm cells were scraped with a pipette tip following 5 min-exposure to 100 μl trypsin-EDTA 0.25%. Cell suspension was vortexed at high speed for 1 min to break up the clumps, and then bacterial counts were assessed by plating serial 10-fold dilutions of the biofilm cell suspension onto selective media MHA plates.

### Motility assays

Swimming, swarming, and twitching motilities were evaluated using dedicated agar media, as previously described (Pompilio et al., 2008). The relative motility of two strains was assessed by using agar prepared with and without 1:2 diluted culture supernatant of the other strain.

### Gene expression assay in mixed biofilm

The effect of *S. maltophilia* RR7 on the transcription levels of several virulence factors (Table 1) of *P. aeruginosa* RR8 was assessed in mixed biofilms by real-time PCR (RT-PCR). *P. aeruginosa* RR8 was cultured as biofilm, both alone and with *S. maltophilia* RR7, in 24-well polystyrene, flat bottom, tissue culture-treated microtiter (BD Falcon), as described above. Following 24 h-incubation at 37°C, biofilms were washed, and then harvested by scraping in Qiazol (Qiagen; Milan, Italy). RNA was then extracted by the phenol-chloroform technique (Kang et al., 2009), treated with DNase I (Applied Biosystems Italia; Monza, Italy), and checked for purity by NanoDrop-2000 spectrophotometer (Thermo Scientific Italia; Milan, Italy). First strand cDNA was synthesized using a High Capacity cDNA reverse transcription kit (Applied Biosystems) according to the manufacturer's protocol. Gene expression was evaluated using a SYBR green (Applied Biosystems) RT-PCR assay. The primers' specificity was assessed both *in silico* with BLAST and by PCR endpoint under the same RT-PCR conditions. Each amplification assay was also tested for *S. maltophilia* RR7 as an additional negative control in each RT-PCR. The ΔΔCt method was used to determine the relative gene expression of each gene in co-culture vs. monoculture biofilms normalized to the expression of the housekeeping gene *proC*.

**Table 1:** **List of primer sequences used in RT-PCR for expression analysis of virulence genes by *P. aeruginosa* RR8 grown as biofilm, both in solo and co-cultured with *S. maltophilia* RR7**.

**Primers, 5′–3′**	**Primer ID**	**Target gene**	**Gene function**	**References**
CTCAGGATGATGGCGATTTC	rhlR-F	*rhlR*	Quorum sensing	Pérez-Osorio et al., 2010
AATTTGCTCAGCGTGCTTTC	rhlR-R	–	–	–
GCGTGCTGCACTACTCCATG	toxA-F	*toxA*	Exotoxin A	Davinic et al., 2009
GTTACCGGCGTTCAGTTCGT	toxA-R	–	–	–
GCTGCGAAACGCTGTTCTTC	vfr-F	*vfr*	Virulence regulator las system	Davinic et al., 2009
GCTGCCGAGGGTGTAGAGG	vfr-R	–	–	–
GCGACCTGGACCTGGGCT	algD-F	*algD*	Enhancer for alginate synthesis	Joly et al., 2005
TCCTCGATCAGCGGGATC	algD-R	–	–	–
GCTTCTGCACGGCAAGGA	lasI-F	*lasI*	Quorum sensing	Joly et al., 2005
ATGGCGAAACGGCTGAGTT	lasI-R	–	–	–
GGCGGATGCGGAAAAGTAC	exoS-F	*exoS*	Type III system effector	Joly et al., 2005
CTGACGCAGAGCGCGATT	exoS-R	–	–	–
TACTCGCTGGGCAAGTTCAGGCG	aprA-F	*aprA*	Alkaline protease	Vettoretti et al., 2009
GTAGCTCATCACCGAATAGGCG	aprA-R	–	–	–
GTACCGGCGTCATGC AGGGTTC	mexC-F	*mexC*	Efflux pump	Savli et al., 2003
TTACTGTTGCGGCGCAGGTGACT	mexC-R	–	–	–
CCAGGACCAGCACGAACT TCT TGC	mexE-F	*mexE*	Efflux pump	Vettoretti et al., 2009
CGACAACGCCAAGGGCGAGTTCACC	mexE-R	–	–	–
CAGGCCGGGCAGTTGCTGTC	proC-F	proC^*^	Proline biosynthesis	Savli et al., 2003
GGTCAGGCGCGAGGCTGTCT	proC-R	–	–	–

* *proC was tested as housekeeping gene*.

### Interpretative criteria and statistical analysis

Each experiment was performed in triplicate and repeated on two different occasions. All statistical analyses were performed by GraphPad Prism software (ver. 4.0; GraphPad Inc, San Diego, USA), considering as statistically significant a *p*-value less than 0.05. Differences were assessed by ANOVA-test followed by Newman-Keuls multiple comparison post-test (kinetics of planktonic growth, adhesion and biofilm formation onto both polystyrene and CFBE41o- cells, and tobramycin activity against preformed biofilms), chi-square test (adhesion and biofilm formation efficiency) or paired Student's *t*-test (motility and gene expression). The efficiency of adhesion and biofilm formation onto CFBE41o- cells was calculated for each strain, both in single and mixed infections, as a percentage referred to the initial inoculum: (A/B) × 100, where A is the number of adhered or biofilm bacteria, and B is the number of bacteria in the initial inoculum.

In mixed cultures, the Competitive Index (CI) was defined as the *S. maltophilia*/*P. aeruginosa* ratio within the output sample divided by the corresponding ratio in the inoculum (input): CI = (*S. maltophilia*/*P. aeruginosa*)_output_/(*S. maltophilia*/*P. aeruginosa*)_input_, where output and input samples were assessed after plating onto MHA serial dilutions of the sample taken at fixed times or the inoculum (*t* = 0), respectively (Macho et al., 2007). For statistical analyses, CI values were first subjected to a Log transformation for normal distribution, then interpreted as follows: a CI value equal to 0 indicates equal competition of the two species; a positive CI value indicates a competitive advantage for *S. maltophilia*; a negative CI value indicates a competitive advantage for *P. aeruginosa*.

Similarly to CI, the Relative Increase Ratio (RIR) was calculated based on the growth results obtained from monocultures of each strain (Macho et al., 2007). Each CI and RIR was analyzed using the Student's *t*-test and the null hypothesis: the mean index was not significantly different from 1.0. When appropriate, CI and RIR from a given experiment were compared using unpaired Student's *t*-test, and significant differences are suggestive of a meaningful competition between the species (Macho et al., 2007).

## Results

### *P. aeruginosa* significantly affects *S. maltophilia* growth in planktonic culture

**Figure 1:**
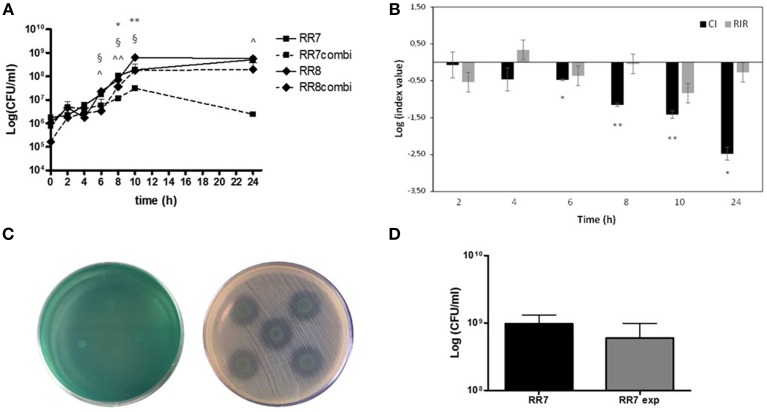
**Kinetics of planktonic growth exhibited by ***S. maltophilia*** RR7 and ***P. aeruginosa*** RR8, considered both as alone and in combination**. *S. maltophilia* RR7 and *P. aeruginosa* RR8 were grown for 24 h in CAMHB in single culture and in co-culture after inoculation at equal ratio from mid-exponential phase pure cultures. Growth rate was monitored by colony count after plating on selective media for both species. **(A)** Growth curves of *S. maltophilia* RR7 and *P. aeruginosa* RR8 strains in pure culture (RR7, RR8) and in co-culture (RR7combi, RR8combi). The results are shown as mean + SD (*n* = 6). ^*^p < 0.05, ^**^p < 0.01, RR7 vs. RR8; ^§^*p* < 0.05, RR8 vs. RR8combi; ^∧^*p* < 0.05, ^∧∧^*p* < 0.01, RR7 vs. RR7combi; ANOVA + Newman-Keuls post-test. **(B)** Competitive index (CI; black bars) and Relative Increase Ratio (RIR; gray bars) calculated from single and dual planktonic cultures of *S. maltophilia* RR7 and *P. aeruginosa* RR8 strains. The results are shown as mean ± SD (*n* = 6). ^*^p < 0.05, ^**^p < 0.01, CI vs. RIR, unpaired *t*-test. **(C)** Evaluation of antibacterial activity using agar spot assay. *S. maltophilia* RR7 vs. *P. aeruginosa* RR8 (left): no antibacterial activity. *P. aeruginosa* RR8 vs. *S. maltophilia* RR7 (right): partial antibacterial activity, as suggested by the regrowth observed within the inhibition zone. **(D)** Transwell assay: *S. maltophilia* RR7 growth without or with (exp) *P. aeruginosa* RR8. The results are expressed as mean + SD (*n* = 6).

The growth curve kinetics of *P. aeruginosa* RR8 and *S. maltophilia* RR7 strains, tested as alone or in mixed culture, were assessed by colony count over 24 h, and the results are shown in Figure 1A. When grown in mixed cultures, the growth kinetics of the two strains were comparable. However, compared with single cultures, the growth of both *S. maltophilia* RR7 and *P. aeruginosa* RR8 in mixed cultures was negatively affected during both log and stationary phases.

To assess further the meaning of the differences between the variations observed in single vs. mixed cultures, CI and RIR were calculated and compared, as shown in Figure 1B. The CI of *S. maltophilia* RR7 vs. *P. aeruginosa* RR8 was significantly different from the respective RIR values between 6 h- and 24 h-incubation, suggesting that *P. aeruginosa* RR8 exerts an inhibitory effect on *S. maltophilia* RR8 growth during both log and stationary growth phases.

### *P. aeruginosa* exhibits antimicrobial activity against *S. maltophilia* in a contact-dependent manner

The agar spot assay results showed that *P. aeruginosa* RR8 is active against *S. maltophilia* RR7, although only when tested as cell suspension (diameter of inhibition zone, mean ± SD: 12.5 ± 1.4 mm), and not as supernatant (Figure 1C). However, the inhibition halo showed a partial regrowth, suggesting that a partial inhibition occurred. Conversely, *S. maltophilia* RR7 showed no activity against *P. aeruginosa* RR8, as supernatant or suspension (Figure 1C).

The inhibitory effect of *P. aeruginosa* RR8 on *S. maltophilia* RR7 was also assessed using a transwell-based assay, and the results are shown in Figure 1D. *S. maltophilia* growth was not significantly affected by the presence of *P. aeruginosa* (9.6 ± 3.4 × 10^8^ CFU/ml vs. 5.9 ± 3.9 × 10^8^ CFU/ml for unexposed and exposed *S. maltophilia*, respectively; *p* > 0.05), which suggests that *P. aeruginosa* inhibits *S. maltophilia* growth in a contact-dependent manner.

### Kinetics of *S. maltophilia* and *P. aeruginosa* interaction during adhesion and biofilm formation onto polystyrene

**Figure 2:**
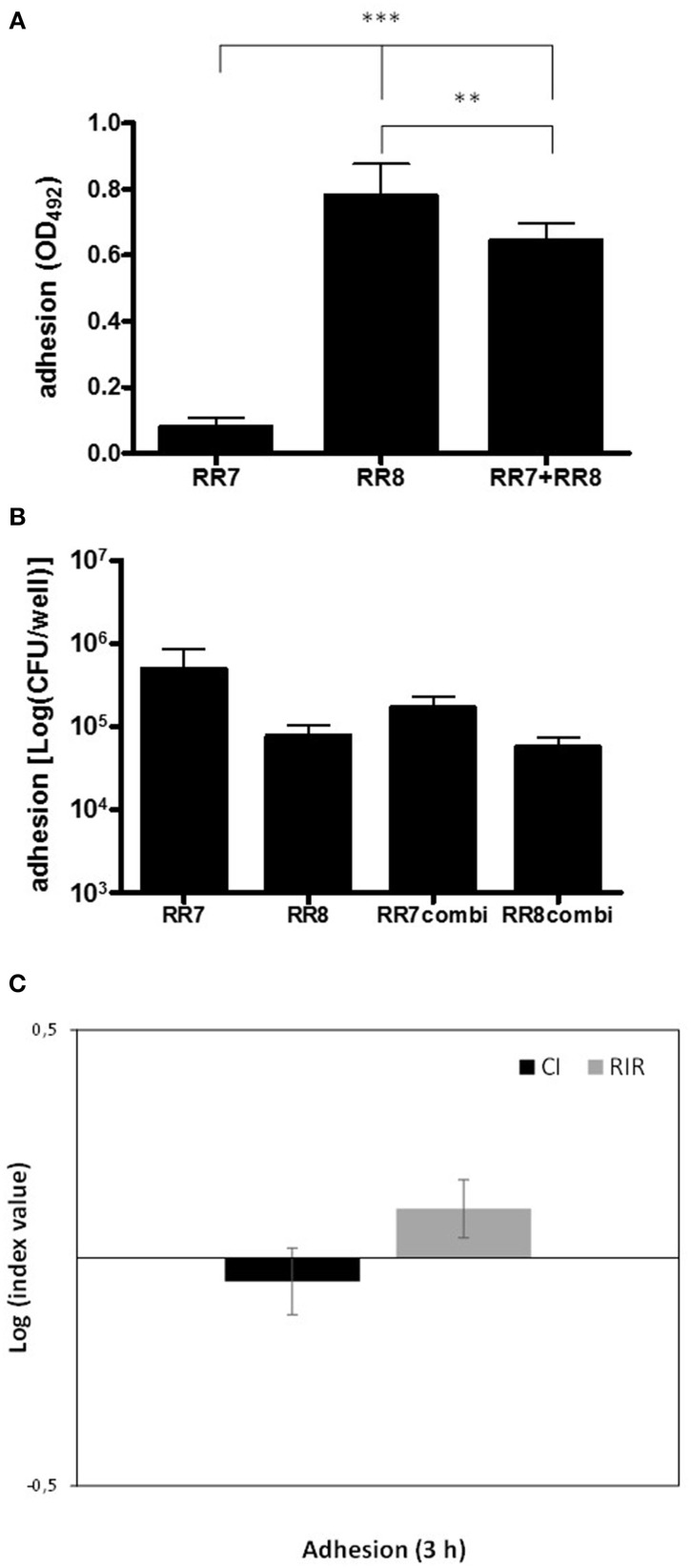
**Kinetics of adhesion onto polystyrene by ***S. maltophilia*** RR7 and ***P. aeruginosa*** RR8, considered both as alone and in combination**. Adhesion was assessed, after 3 h of incubation at 37°C, both by crystal violet and viable count assays. **(A)** Crystal violet assay. *S. maltophilia* RR7, and *P. aeruginosa* RR8 were tested both alone (RR7, RR8), and in mixed infection (RR7 + RR8), and the results are shown as mean + SD (*n* = 6). ^**^p < 0.01, ^***^p < 0.001, ANOVA + Newman-Keuls post-test. **(B)** Viable count assay. *S. maltophilia* RR7 and *P. aeruginosa* RR8 strains were tested both alone (RR7, RR8), and in mixed infection (RR7combi, RR8combi), and the results are shown as mean + SD (*n* = 6). No statistically significant differences were found among groups by ANOVA + Newman-Keuls post-test. **(C)** Competitive index (CI; black bars) and Relative Increase Ratio (RIR; gray bars), calculated from single and dual cultures of *S. maltophilia* RR7 and *P. aeruginosa* RR8. The results are shown as mean ± SD (*n* = 6). CI vs. RIR, no statistically significant difference, unpaired *t*-test.

The interaction during adhesion phase between *S. maltophilia* RR7 and *P. aeruginosa* RR8 strains was assessed, tested as single and mixed cultures, after 3 h-incubation, both by crystal violet and the viable count assays; the results are reported in Figure 2.

The crystal violet assay showed that *P. aeruginosa* RR8, when cultured alone, exhibited a significantly higher adhesiveness than *S. maltophilia* RR7 (OD_492_, mean ± SD: 0.781 ± 0.095 vs. 0.079 ± 0.026, respectively; *p* < 0.001) (Figure 2A). No differences, however, were found between the strains in terms of cell viability (Figure 2B), which suggests that extracellular polymeric substance (EPS) contributes substantially to *P. aeruginosa* RR8 adhesion.

In mixed infections, the numbers of viable cells of each strain that adhered to polystyrene were not affected by the presence of the other (Figure 2B), as was confirmed by comparable CI and RIR values (Figure 2C). However, in mixed culture the biomass adhesion level of *S. maltophilia* RR7 was significantly lower than that of *P. aeruginosa* RR8 (OD_492_, mean ± SD: 0.645 ± 0.051 vs. 0.781 ± 0.095, respectively; *p* < 0.01), which indicates that the presence of *S. maltophilia* RR7 leads to a reduction in *P. aeruginosa* RR8 EPS.

**Figure 3:**
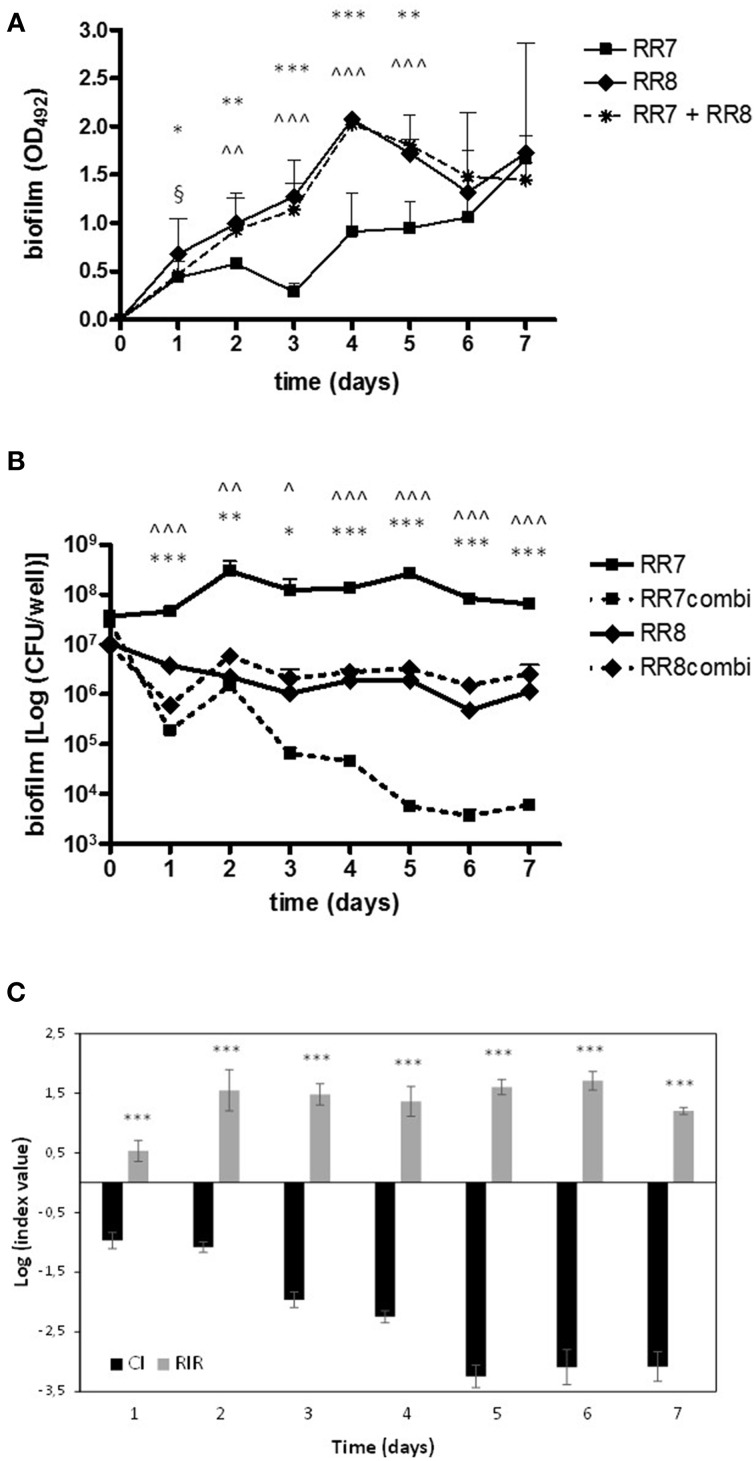
**Kinetics of biofilm formation onto polystyrene by ***S. maltophilia*** RR7 and ***P. aeruginosa*** RR8, both considered as alone and in combination**. Biofilm formation was assessed, over 7 days, both by **(A)** crystal violet and **(B)** viable count assays. *S. maltophilia* RR7 and *P. aeruginosa* RR8 were tested both alone (RR7, RR8), and in mixed infection (RR7 + RR8), and the results are shown as mean + SD (*n* = 6). ^*^p < 0.05, ^**^p < 0.01, ^***^p < 0.001, RR7 vs. RR8; ^∧^*p* < 0.05, ^∧∧^*p* < 0.01, ^∧∧∧^*p* < 0.001, RR7 vs. RR7combi; ^§^*p* < 0.05, RR8 vs. RR8combi; ANOVA + Newman-Keuls post-test. **(C)** Competitive index (CI; black bars) and Relative Increase Ratio (RIR; gray bars) calculated from single and dual biofilm cultures of *S. maltophilia* RR7 and *P. aeruginosa* RR8. The results are shown as mean ± SD (*n* = 6). ^***^p < 0.001, CI vs. RIR, unpaired *t*-test.

The interaction in biofilm growth between *S. maltophilia* RR7 and *P. aeruginosa* RR8, tested as single and mixed cultures, was monitored over 7 days, by both crystal violet and viable count assays; results are shown in Figure 3.

The kinetics of biofilm formation, as assessed by crystal violet staining, are summarized in Figure 3A. In monoculture, from day 1 until day 5 of incubation *P. aeruginosa* RR8 formed significantly higher biofilm biomass amount than *S. maltophilia* RR7. The kinetics of biofilm formation by *P. aeruginosa* RR8 and RR7+RR8 mixed infection followed a comparable trend throughout the 7 day period. From day 2 until day 5 of incubation, the mean biofilm biomass amount formed by both *P. aeruginosa* RR8 and mixed infection was significantly higher than when *S. maltophilia* RR7 was cultured alone. On day 6, all conditions tested produced comparable mean biofilm biomass values.

The biofilm formation kinetics evaluated by the viable count are shown in Figure 3B. In monoculture, the viability of biofilm formed by *S. maltophilia* RR7 was higher than that of *P. aeruginosa* RR8 over the entire period (Figure 3B). Given the different endpoints measured by the crystal violet (biofilm biomass, consisting of both EPS and cells) and the viable count (biofilm viability) assays, our results indicate that only in *P. aeruginosa* does the amount of EPS increase over time during biofilm formation, as is also confirmed by the mucoid appearance of the biofilm samples observed during macroscopic analysis of the 96-well plate.

When *S. maltophilia* RR7 was co-cultured with *P. aeruginosa* RR8 in mixed biofilms, its viability was significantly decreased throughout the period. Conversely, the biofilm viability of *P. aeruginosa* RR8 was not affected by the presence of *S. maltophilia* RR7.

The CI values of *S. maltophilia* vs. *P. aeruginosa* were always significantly different from the RIR values (*p* < 0.001) throughout the 7 days of incubation, which suggests that *P. aeruginosa* outcompetes *S. maltophilia* affecting its growth in mixed biofilms as well as in planktonic cultures (Figure 3C).

### Adhesion to and biofilm formation on CFBE41o- cells

**Figure 4:**
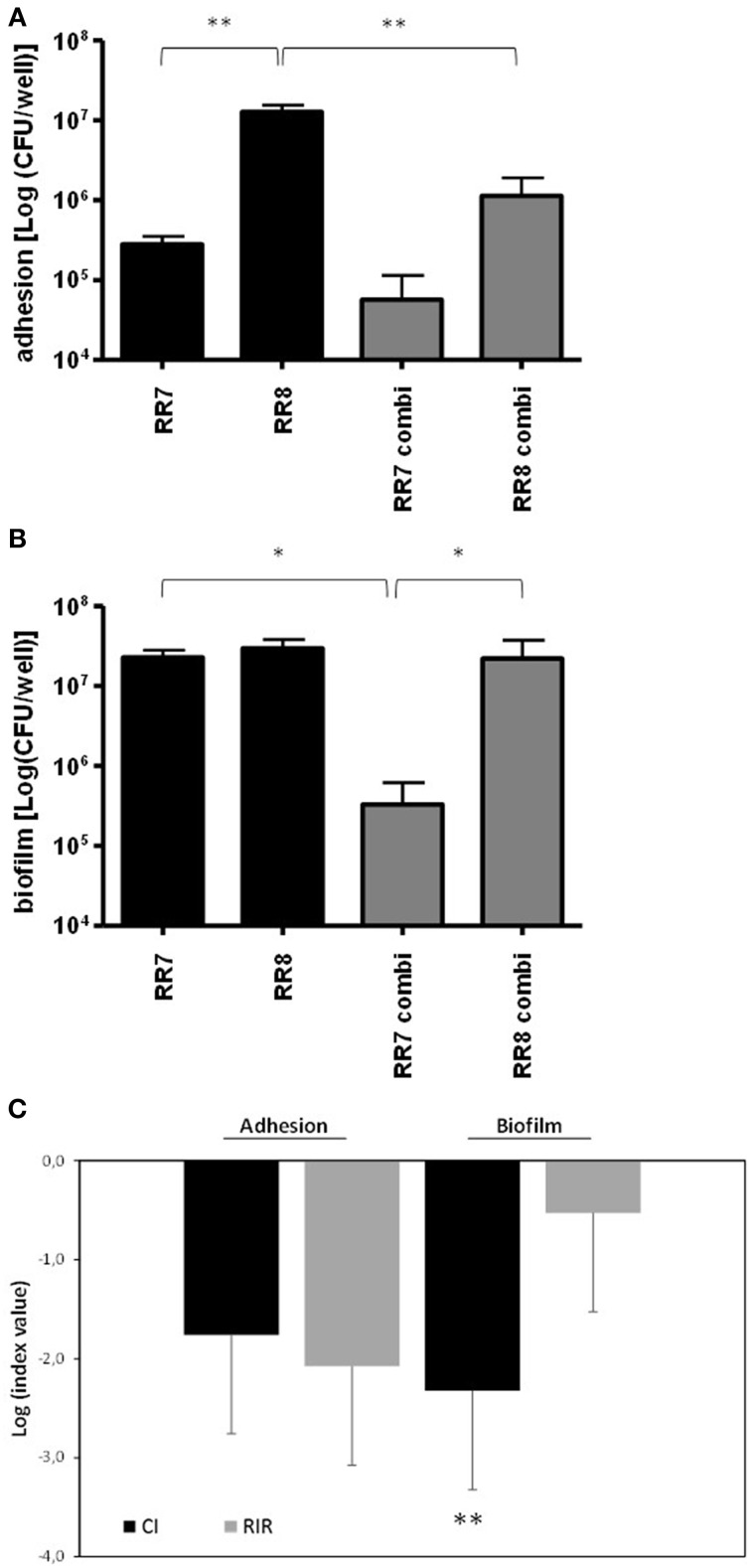
**Adhesion and biofilm formation by ***S. maltophilia*** RR7 and ***P. aeruginosa*** RR8 onto CFBE41o- CF bronchial cells**. CFBE41o- cell monolayers were exposed for **(A)** 3 h (adhesion assay) or **(B)** 24 h (biofilm formation assay) to *S. maltophilia* RR7 and *P. aeruginosa* RR8 (each at 10^6^ CFU/ml; MOI: 10), tested as alone (RR7, RR8) or in mixed cultures (RR7combi, RR8combi). ^*^p < 0.05, ^**^p < 0.01, ANOVA + Newman-Keuls post-test. **(C)** In mixed cultures, the Competitive Index (CI) and the Relative Increase Ratio (RIR) were calculated as described in Materials and Methods. The results are shown as mean + SD (*n* = 6). ^**^p < 0.01; unpaired *t*-test.

*P. aeruginosa* RR8 and *S. maltophilia* RR7 were evaluated, both alone and in mixed culture, for adhesion to, and biofilm formation onto, CFBE41o- CF cell monolayer. The results of the viable count assay are summarized in Figure 4.

When monolayers were separately infected, the adhesiveness of *P. aeruginosa* RR8 to CFBE41o- cells was significantly higher than that of *S. maltophilia* RR7 (1.3 ± 0.3 × 10^7^ vs. 2.8 ± 0.7 × 10^5^ CFU/well, respectively; *p* < 0.01) (Figure 4A). Conversely, when CFBE41o- cell monolayer was concomitantly challenged by both strains their degrees of adhesiveness were comparable. However, the adhesiveness of *P. aeruginosa* RR8 was significantly less than that observed in monoculture (1.1 ± 0.7 × 10^6^ vs. 1.3 ± 0.3 × 10^7^ CFU/well, respectively; *p* < 0.05), unlike *S. maltophilia* RR7, whose adhesiveness was not affected by the presence of *P. aeruginosa* RR8. The comparative analysis of the CI and the RIR values showed no statistically significant difference (Figure 4C), although a comparison of efficiency values, from single and mixed cultures, showed a significant decrease for *P. aeruginosa* RR8 (from 12.9 to 4.9%, respectively; *p* < 0.05), whereas a significant increase was found for *S. maltophilia* RR7 (from 0.08 to 0.3%, respectively; *p* < 0.001).

Both *S. maltophilia* RR7 and *P. aeruginosa* RR8 showed comparable biofilm viability when tested in monoculture (Figure 4B). However, when CFBE41o- cell monolayer was simultaneously infected by both strains, the concentration of *P. aeruginosa* RR8 in mixed biofilm was significantly higher than that of *S. maltophilia* RR7 (2.2 ± 1.5 × 10^7^ vs. 3.2 ± 2.9 × 10^5^ CFU/well, respectively; *p* < 0.05), whose concentration was significantly lower than that observed in monomicrobial biofilm (2.3 ± 0.5 × 10^7^ CFU/well; *p* < 0.05) (Figure 4B).

The comparative analysis of the CI and the RIR values obtained for biofilm formation assay showed statistically different values (−2.3 ± 0.4 vs. −0.5 ± 0.4, respectively; *p* < 0.01) (Figure 4C). Relative to the biofilm cells/initial inoculum ratio calculated for each strain, the values obtained from mono- and mixed cultures were significantly elevated for *P. aeruginosa* RR8 (from 31.8 to 96.2%, respectively; *p* < 0.001), while a significant decrease was observed for *S. maltophilia* RR7 (from 6.9 to 0.5%, respectively; *p* < 0.001).

Taken together, these results suggest the existence of a reciprocal interference between these two species in the adhesion step. *S. maltophilia* RR7 negatively affects *P. aeruginosa* RR8 adhesiveness, whereas later, during biofilm formation onto CFBE41o- cells, *P. aeruginosa* RR8 outcompetes *S. maltophilia* RR7, probably by inhibiting its growth.

### Exposure to *S. maltophilia* culture supernatant significantly reduces swimming motility in *P. aeruginosa*

**Figure 5:**
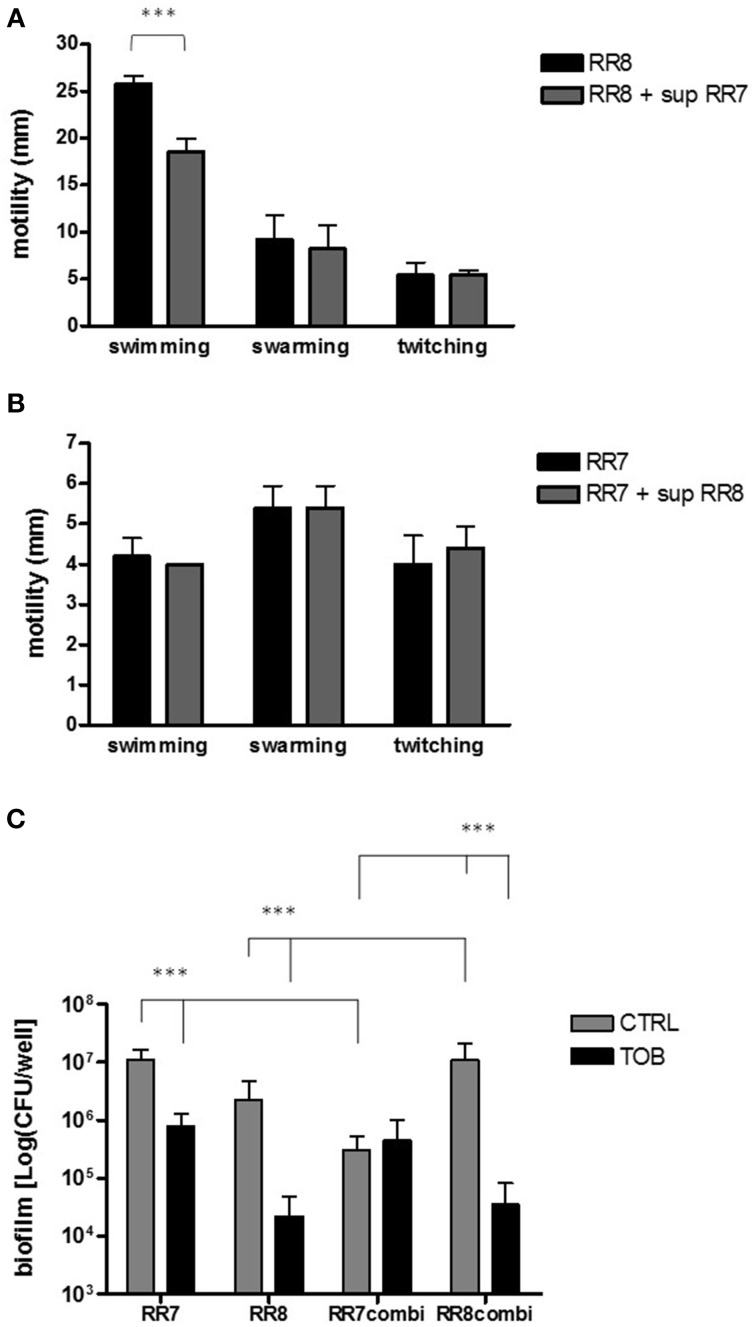
**(A,B)** Effect of culture supernatants on motility. Swimming, swarming, and twitching motilities of each strain were assessed as previously described (Pompilio et al., 2008). **(A)** *P. aeruginosa* RR8 motility was evaluated both in the presence and absence of *S. maltophilia* RR7 culture supernatant (sup RR7). **(B)** *S. maltophilia* RR7 motility was evaluated both in the presence and absence of *P. aeruginosa* RR8 culture supernatant (sup RR8). The results are shown as mean + SD (*n* = 6). ^***^p < 0.001, paired-*t* test. **(C)** Activity of tobramycin against preformed biofilms. Monomicrobial and mixed biofilms were allowed to grow for 24 h, and then they were exposed to tobramycin 128 μg/ml (TOB) or CAMHB only (control, CTRL) for further 24 h. Biofilm's viability was then assessed by viable count. The results are shown as mean + SD (*n* = 6). ^***^p < 0.001, ANOVA + Newman-Keuls post-test.

The effect of the presence of one strain, tested as culture supernatant, on swimming, swarming and twitching motilities of the other strain was evaluated and the results are summarized in Figures 5A,B.

The swimming motility exhibited by *P. aeruginosa* RR8 was significantly reduced by exposure to *S. maltophilia* RR7 culture supernatant (mean ± SD: 18.6 ± 1.3 vs. 25.8 ± 0.8 mm, with or without supernatant, respectively; *p* < 0.001) (Figure 5A). No significant variations were observed between groups for swarming and twitching motilities. *P. aeruginosa* RR8 supernatant did not affect any of *S. maltophilia* RR7 motility types tested (Figure 5B).

### *S. maltophilia* is less susceptible than *P. aeruginosa* to tobramycin in mixed biofilms

The activity of tobramycin at 128 μg/ml against single and mixed preformed biofilm was assessed by the viable count and the results are summarized in Figure 5C. Exposure to tobramycin significantly reduced the viability of both *S. maltophilia* RR7 and *P. aeruginosa* RR8 monomicrobial biofilms (*S. maltophilia* RR7: 1.1 ± 0.5 × 10^7^ vs. 8.0 ± 5.0 × 10^5^ CFU/well; *P. aeruginosa* RR8: 2.2 ± 2.5 × 10^6^ vs. 2.2 ± 2.4 × 10^4^ CFU/well; unexposed and tobramycin-treated biofilms, respectively; *p* < 0.001). However, when mixed biofilms were tested, tobramycin resulted to be effective against *P. aeruginosa* RR8 only (1.1 ± 0.9 × 10^7^ vs. 3.4 ± 4.7 × 10^4^ CFU/well; unexposed and tobramycin-treated biofilms, respectively; *p* < 0.001).

### *S. maltophilia* significantly affects *P. aeruginosa* virulence in mixed biofilm

**Figure 6:**
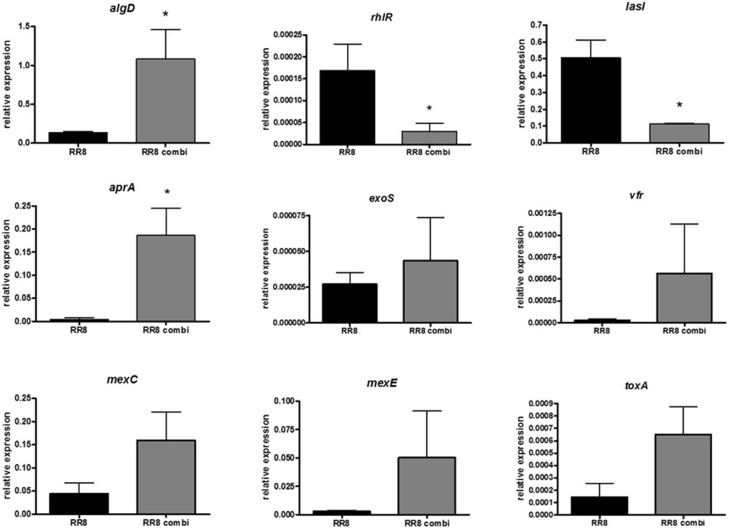
**Effect of ***S. maltophilia*** RR7 on ***P. aeruginosa*** RR8 virulence-related gene expression in mixed biofilm**. *S. maltophilia* RR7 and *P. aeruginosa* RR8 were allowed to grow, as mixed biofilm, in a 24-well microtiter polystyrene plate at 37°C for 24 h (RR8 combi, gray bars). Relative expression of nine selected *P. aeruginosa* virulence genes (*algD, rhlR, lasI, aprA, exoS, vfr, mexC, mexE, toxA*) were then measured by RT-PCR. *P. aeruginosa* grown alone as biofilm was considered as control (RR8, black bars). The results are shown as mean + SD (*n* = 6). ^*^p < 0.05, paired *t*-test.

The effect of exposure to *S. maltophilia* RR7 on the expression of nine selected virulence genes (*rhlR, lasI, aprA, vfr, exoS, toxA, algD, mexC, mexE*) of *P. aeruginosa* RR8 was assayed, by RT-PCR, both in mono- and co-cultured biofilms; the results are summarized in Figure 6. When grown in mixed biofilm with *S. maltophilia* RR7, *P. aeruginosa* RR8 significantly overexpressed *aprA*, and *algD* genes (*p* < 0.05), codifying for protease and alginate, respectively, whereas the QS-related *rhlR* and *lasI* genes were down-regulated (*p* < 0.05). The presence of *S. maltophilia* RR7 also caused a considerable increase of efflux pump systems-related *mexC* and *mexE* genes, as well as of *toxA*, codifying for exotoxin A, expression by *P. aeruginosa* RR8, although this trend resulted not to be statistically significant, probably due to high SD values.

## Discussion

The originality of this study is two-fold. Firstly, it evaluates the nature of interactions occurring between *P. aeruginosa* and *S. maltophilia* under both planktonic and biofilm growth, and specifically whether these interactions offer enhanced fitness compared to single cultures. Secondly, it considers *S. maltophilia* and *P. aeruginosa* strains isolated at the same time from the same CF patient during a PE episode, whereas previous works were conducted using strains isolated from different, and non-CF, patients (Kataoka et al., 2003; Ryan et al., 2008; Varposhti et al., 2014).

Following examination of the interaction between *S. maltophilia* and *P. aeruginosa* under planktonic growth conditions, the CI and RIR values suggested that *P. aeruginosa* outcompetes *S. maltophilia*, during both exponential and stationary growth phases. This microbial antagonism could be due to competition for both nutrients and space or, more directly, to an antibacterial effect of one species toward the other (Harrison, 2007; Hibbing et al., 2010). Our findings from the agar spot assay in fact clearly showed, for the first time, that *P. aeruginosa* exerts antibacterial activity against *S. maltophilia*. As suggested by the transwell-based assay results, this effect is probably not mediated by exoproducts, but requires direct contact between cells, which suggests that *P. aeruginosa*, in order to achieve new niches, might kill *S. maltophilia* by injecting specific proteins via the type VI secretion system (Tashiro et al., 2013).

Despite the increasing interest in the crucial role played by biofilm in determining CF infections, interspecies interactions in mixed biofilms are still poorly understood. Using a polystyrene microtiter assay, the reciprocal interaction between *S. maltophilia* and *P. aeruginosa* was initially evaluated at the adhesion phase, the first step of biofilm formation. Our findings confirmed that EPS plays a crucial role during *P. aeruginosa* adhesion, and interestingly showed that the presence of *S. maltophilia* reduces the amount of EPS, probably secondary to loss or a reduced synthesis, therefore affecting *P. aeruginosa* adhesion to the substratum.

The kinetics of biofilm formation were then monitored throughout the 7 day-incubation period with daily medium replacement, in order to simulate a chronic infection. When both species were cultured alone, we found that in *P. aeruginosa*, unlike *S. maltophilia*, EPS amount increases over time during biofilm formation. When simultaneously cultured in mixed biofilms, we observed that *S. maltophilia* and *P. aeruginosa* cannot coexist in a dynamic equilibrium in mixed biofilm: *S. maltophilia* will always be outcompeted. The results from the viable count in fact indicated that the contribution of *S. maltophilia* to the biomass of mixed biofilm is negligible, being at least 100-fold lower than *P. aeruginosa* concentration. The comparative evaluation between single and dual specie biofilms showed that the capacity of *P. aeruginosa* to form biofilms is unaffected by the presence of *S. maltophilia*, while that of *S. maltophilia* is significantly reduced in the presence of *P. aeruginosa*. The antibacterial activity exhibited in planktonic cultures by *P. aeruginosa* is probably one of the mechanisms that inhibits *S. maltophilia* biofilm formation.

In contrast with our findings, Varposhti et al. (2014) found that *S. maltophilia*, in mixed cultures, induces *P. aeruginosa* biofilm biomass formation on Foley catheter. The discrepancy could be explained by the fact that strains tested in combinations by Varposhti et al. were from different patients with polymicrobial lower respiratory infections. This might be particularly relevant in the case of CF chronic infections, where *P. aeruginosa* undergoes adaptation to the CF lung leading to patho-adaptative lineages genotypically and phenotypically different (Huse et al., 2010), which has been observed not only when comparing isolates from different CF patients, but also when comparing multiple isolates from the same CF respiratory specimen (Lee et al., 2005).

The polystyrene microtiter assay is classically used for biofilm studies due to its ease of use and ability for high-throughput testing. However, since not all variables can be controlled, the conditions still differ significantly from those observed during natural infections and, therefore, the results might be of limited clinical relevance. The interaction between *P. aeruginosa* and *S. maltophilia* during adhesion and biofilm formation was therefore also assessed using a CF-derived bronchial epithelial cell monolayer.

In single infections, *P. aeruginosa* was more adhesive than *S. maltophilia*, which disagrees with the polystyrene microtiter assay. However, when the cell monolayer was simultaneously challenged with mixed cultures, the adhesiveness of *P. aeruginosa* was significantly reduced relative to monoculture, while that of *S. maltophilia* remained unaffected. The results from motility assays suggested that *S. maltophilia* might reduce *P. aeruginosa* adhesiveness to bronchial cells by affecting its ability to reach the substratum via swimming motility, probably as a consequence of flagellar loss or a reduced expression of flagella-associated genes.

In accordance with the polystyrene microtiter assay, the viable count showed that *P. aeruginosa* outcompetes *S. maltophilia* in mixed biofilms formed onto CF bronchial cells. Accordingly, we also observed that *S. maltophilia* can not affect *P. aeruginosa* biofilm formation by modulating its swarming or twitching motility.

Recent studies on the behavior of multispecies communities have shown that the virulence and the gene expression of pathogens can be modulated by the presence of other bacterial species (Duan et al., 2003). Therefore, we performed a comparative analysis of transcript levels of several *P. aeruginosa* virulence factors both in single- and dual-species biofilms. The quantitative PCR results suggested that *S. maltophilia* changes the physiology and, consequently, the virulence of *P. aeruginosa*. Specifically, both *aprA* and *algD* operon—codifying for the frank *P. aeruginosa* virulence factors alkaline protease and alginate, respectively—were up-regulated in the presence of *S. maltophilia*, whereas the QS-related *rhlR* and *lasI* genes were down-regulated. These differences may be the expression of a defensive reaction by *P. aeruginosa* to the presence of *S. maltophilia*. Alternatively, since both bacterial species exist ubiquitously in various environments, including the rhizosphere of plants (Berg et al., 2005), the lack of significant differences in the expression of other genes observed in co-culture may be highly suggestive of their relative acclimatization to each other.

*P. aeruginosa* causes severe tissue damage by the expression of the alkaline protease AprA, especially in CF patients where it has been associated with increased infectivity and virulence, suggesting a role in processes related to bacterial colonization and/or exacerbation in the CF lung (Burke et al., 1991; Kim et al., 2006). Our findings suggested that in mixed infections *S. maltophilia* might indirectly facilitate the onset of PEs in CF patients by inducing increased proteolytic activity in *P. aeruginosa*, probably via a Las/RhI independent pathway.

On the other hand, *S. maltophilia* might also promote *P. aeruginosa* adaptation to the airways of CF patients by favoring phenotypic traits acquired by *P. aeruginosa* only during chronic infections, which has the result of promoting long-term survival in CF lungs. Particularly, we observed in mixed biofilms the loss of LasR function and overproduction of alginate. Loss of QS regulation is generally considered a hallmark of chronic virulence and has been described for several *P. aeruginosa* CF isolates (Geisenberger et al., 2000; D'Argenio et al., 2007). Specifically, loss of LasR function has clinical implications for disease progression and antibiotic resistance since it produces an increase in β-lactamase activity, which augments tolerance to ceftazidime, a widely used β-lactam antibiotic in CF patients (D'Argenio et al., 2007). In addition, mucoid strains overproducing alginate have been found to be dominant in chronic lung infections, and therefore correlated with a poor prognosis (Yang et al., 2011). Furthermore, by increasing *algD* expression *S. maltophilia* might favor the protection of *P. aeruginosa* against oxygen radicals from activated PMNs (Govan and Deretic, 1996), and improve its ability to form more robust biofilms (Hoffmann et al., 2005).

Understanding the interspecies interactions is essential, not only because they can potentially modulate the virulence and the persistence of pathogens such as *P. aeruginosa*, but also because it might lead to the development of new, more effective, therapeutic strategies. We found that the exposure of a mixed biofilm to tobramycin reduced the viability of *P. aeruginosa* but not of *S. maltophilia*. It is, therefore, plausible that the *S. maltophilia*-induced alginate expression by *P. aeruginosa* might ensure added protection both to *S. maltophilia* against tobramycin, and to *P. aeruginosa* against other antimicrobial agents. This might explain why about 25% of patients do not recover their baseline lung function after treatment for a PE episode (Sanders et al., 2010, 2011), therefore suggesting that a suitable CF treatment should take into consideration the threat of such nosocomial indirect pathogens as *S. maltophilia*.

In conclusion, for the first time our findings show that a reciprocal interference between *S. maltophilia* and *P. aeruginosa* in CF lung is plausible. *P. aeruginosa* exhibits an aggressive lifestyle dominating *S. maltophilia* during competitive planktonic growth and biofilm development. However, in CF lung *S. maltophilia* cannot be considered as simply a “by-stander.” In fact, growing in a biofilm together with *S. maltophilia* changes the physiology of *P. aeruginosa* and, therefore, modulates its virulence profile. This might confer some selective “fitness advantage” to *P. aeruginosa* under the specific conditions of chronic infection or, alternatively, cause its hypervirulentation thus leading to PE.

Our findings, however, need to be confirmed by *in vitro* and *in vivo* investigations focused on other CF *S. maltophilia/P. aeruginosa* combinations since, in infections where both species are present, the outcome over time could be highly influenced by the phenotype of the strains involved.

## Author contributions

AP performed analyses, statistically evaluated results and, together with GDB, drafted the manuscript and assisted in the study design. VC and SDN performed analyses. FV designed and supervised the analyses of gene expression. EF collected and processed clinical specimens, and provided clinical expertise for the discussion of the results. GDB supervised analyses, defined the study design, discussed the results and drafted the manuscript. All authors read, reviewed, and approved the final manuscript.

### Conflict of interest statement

The Reviewer Jose L. Martinez declares that, despite co-hosting a Research Topic with Giovanni Di Bonaventura, the review process was handled objectively and no conflict of interest exists. The authors declare that the research was conducted in the absence of any commercial or financial relationships that could be construed as a potential conflict of interest.

## Abbreviations

CF: cystic fibrosis
PE: pulmonary exacerbation
QS: quorum sensing
MHA: Mueller-Hinton agar
TSB: tryptone soya broth
CAMHB: cation-adjusted Mueller-Hinton broth
PBS: phosphate buffered saline
OD: optical density
EDTA: ethylenediaminetetraacetic acid
MOI: multiplicity of infection
RT-PCR: real-time PCR
CI: competitive index
RIR: relative increase ratio
EPS: extracellular polymeric substance.
